# Third-Generation Trabecular Micro-Bypass Implantation and Phacoemulsification in Patients with Glaucoma: A Multicenter Study

**DOI:** 10.3390/vision9030061

**Published:** 2025-07-19

**Authors:** Mitchell Shultz, Zachary M. Vest, Valerie Trubnik, Steven R. Sarkisian, Dana M. Hornbeak

**Affiliations:** 1 Shultz-Chang Vision, 18350 Roscoe Blvd #101, Northridge, CA 91325, USA; 2 Mile High Eye Institute, Sheridan, CO 80110, USA; 3 Rocky Vista University, Englewood, CO 80112, USA; 4 Ophthalmic Consultants of Long Island, Manhasset, NY 11030, USA; valerietrubnik@yahoo.com; 5 Oklahoma Eye Surgeons, PLLC, Oklahoma City, OK 73112, USA; drsarkisian@okeyesurgeons.com; 6 Glaukos Corporation, Aliso Viejo, CA 92656, USA; dana.hornbeak@gmail.com

**Keywords:** glaucoma, microinvasive glaucoma surgery (MIGS), iStent infinite, cataract, intraocular pressure, multi-center, third generation, trabecular micro-bypass

## Abstract

This multicenter study evaluated the effectiveness and safety of third-generation trabecular micro-bypass implantation (iStent^®^ infinite) combined with phacoemulsification (n = 233 eyes). Key outcomes through 12 months included the mean change in intraocular pressure (IOP) and the number of topical medications, as well as proportions achieving IOPs ≤ 18/15/12 mmHg or using 0/1/2/ ≥ 3 medications. In all eyes with 12-month follow-up data (n = 96, consistent cohort), the mean IOP reduced from 17.2 ± 4.2 mmHg preoperatively to 13.8 ± 3.0 mmHg at Month 12 (*p* = 0.001), while the mean number of medications reduced from 1.24 ± 0.91 preoperatively to 0.61 ± 0.96 at Month 12 (*p* = 0.001). The proportions of eyes achieving IOP ≤ 18/15/12 mmHg increased from 63.5%, 34.4%, and 14.6% preoperatively to 92.7%, 71.9%, and 37.5%, respectively at Month 12, (all *p* = 0.001). The proportions of eyes off medication increased from 16.7% preoperatively to 62.5% at Month 12 (*p* = 0.001). This study provides clinically relevant, real-world results that demonstrate significant reductions in IOP and the number of topical glaucoma medications required following iStent infinite trabecular micro-bypass and phacoemulsification.

## 1. Introduction

Globally, glaucoma remains a leading cause of blindness, estimated in 2020 to be the source of permanent vision loss in 3.6 million individuals over age 50 and projected to affect over 111 million people by 2040 [1,2]. If untreated, glaucoma results in progressive, permanent, and inevitable vision loss. Lowering intraocular pressure (IOP) has been well-established as the key objective in management, reducing the progression of ocular hypertension and open-angle glaucoma (OAG). IOP reduction by topical pharmacotherapy has been the foundation of glaucoma treatment for decades, demonstrating efficacy in delaying the progression of visual field loss with a reasonable safety profile. The limitations of topical therapy in glaucoma management are well known, as they can be associated with substantial side effects, cause damage to the ocular surface, and result in an ongoing financial burden and high rates of patient nonadherence, compromising effectiveness [3,4,5].

Early procedural interventions, such as filtering surgery by trabeculectomy and tube shunts, were often offered as a reliably effective solution for patients unsuccessfully managed with topical therapy, but were also accompanied by a higher risk of serious complications. Within the past two decades, glaucoma management has shifted toward more minimally invasive advancements, including selective laser trabeculoplasty (SLT), minimally invasive glaucoma surgery (MIGS), and sustained-release procedural pharmaceuticals. There has been widespread uptake of MIGS procedures, becoming well-recognized as a surgical intervention for all severities of glaucoma, delivering effective IOP control with less risk, and allowing for potential intervention at an earlier stage in the disease. Recently, a review of data from the American Academy of Ophthalmology IRIS Registry reported that, of over 350,000 eyes receiving glaucoma surgeries in the United Stated between 2013 and 2020, 70% were MIGS, with the iStent implant (Glaukos Corp., San Clemente, CA, USA) being by far the most common (64%) [6].

The iStent^®^ trabecular micro-bypass (containing one stent) was the first MIGS device to be approved by the United States Food and Drug Administration (FDA), followed by the next-generation iStent^®^ *inject* (containing two stents) and, recently, the iStent^®^ infinite (three stents). All three iterations of iStent apply the same physiological mechanism: creating a patent pathway to bypass the trabecular meshwork, facilitating direct aqueous flow into Schlemm’s canal, thereby reducing IOP. Clinical studies conducted across the iStent models have demonstrated significant reductions in IOP and medication burden and a favorable safety profile [7,8,9,10,11,12,13,14,15,16]. The pivotal study of iStent infinite included 72 eyes with a pre-operative medicated (mean 3.1 medications) IOP of 23.4 mmHg. At 12 months following iStent infinite implantation, 76.1% met the responder endpoint of ≥20% IOP reduction on the same or fewer ocular hypotensive medication classes as baseline. The mean IOP reduction was 5.9 mmHg vs. baseline, and 53.0% of eyes achieved ≥30% reduction [12]. The safety profile was favorable, with no explants, infections, or device-related interventions or hypotony. Recently, the INTEGRITY study was a prospective randomized controlled trial of eyes with OAG that underwent the standalone implantation of iStent infinite or Hydrus^®^ Microstent (Alcon) [17]. The study showed that at 6 months postoperatively, the difference between the groups was statistically significantly different (78.2% iStent infinite versus 65.0% Hydrus) for the predetermined primary effectiveness endpoint, with an unmedicated mean diurnal IOP reduction ≥20% from baseline in eyes with no surgical complications [17].

In addition to these standalone studies, Vest et al. published a retrospective analysis of a large real-world dataset of eyes with mild to moderate primary open-angle glaucoma (POAG) patients who underwent iStent infinite implantation and phacoemulsification [15]. At 12 months postoperative vs. preoperative, they found statistically significant reductions in the mean IOP and medications, with IOP reducing from 18.1 mmHg to 13.8 mmHg and the mean medications reducing from 1.38 to 1.06 medications (both *p* < 0.05) [15].

Further real-world studies, encompassing various clinical sites and a spectrum of glaucoma subtypes and severities, will provide valuable evidence to surgeons to support management decisions. Currently, a need remains for this type of real-world evidence, outside the restrictive structure of a clinical trial. Herein, we report outcomes from a US-based multicenter series of glaucoma patients that underwent iStent infinite implantation combined with phacoemulsification. To our knowledge, this constitutes the first real-world multicenter publication of iStent infinite with cataract surgery in the literature to-date.

## 2. Materials and Methods

### 2.1. Study Design and Participants

This was a retrospective multicenter study of eyes undergoing iStent infinite implantation in combination with phacoemulsification. Consecutive cases of eyes with documented glaucoma and cataract undergoing iStent infinite implantation and phacoemulsification by four surgeons at four surgical sites in the United States between December 2022 and October 2024 were reviewed. Data were collected, pooled and analyzed in accordance with the tenets of the Declaration of Helsinki. All patients gave informed consent before undergoing surgery. The study was reviewed and approved by the WCG Institutional Review Board (#20250869).

The primary study outcomes were reductions in the mean IOP and medications from the preoperative period through Month 12 postoperatively. Additional outcomes included the proportion of eyes with IOP ≤ 12 mmHg, ≤15 mmHg and ≤18 mmHg, and the proportion of eyes using 0, 1, 2, 3 or more topical medications. Since eyes had different durations of follow-up, analyses were completed in the observed cohort (all available eyes at each visit) and the consistent cohort (eyes with data at both Baseline and 12 months).

Eligible patients were ≥18 years old and had a history of cataract necessitating removal, and a need for reduction in IOP and/or medications. All glaucoma severities were included (mild, moderate, severe), following ICD 10 definitions of severity [mild, no visual field (VF) change; moderate, VF change in one hemifield but not within 5 degrees of fixation; and severe, VF change in two hemifields or within 5 degrees of fixation]. Exclusion criteria consisted of ocular inflammation, eye surgery in the past 3 months, or clinical parameters that, according to the surgeon, would make them ineligible for either stent implantation or phacoemulsification, such as angle closure or corneal pathology preventing gonioscopy.

### 2.2. Surgical Procedure

The sequence of the surgical procedure was as follows. Each patient underwent standard phacoemulsification cataract extraction and intraocular lens implantation. Following cataract surgery, iStent infinite implantation was completed. The iStent infinite Trabecular Micro-Bypass System and implantation method have been previously described [12,17]. The iStent infinite system has three preloaded intraocular stents manufactured from implant grade titanium. Each of the three stents is 360 µm in both length and flange diameter, with a central inlet and four outlet lumens of 80 µm in diameter, providing up to 240° of collector channels to deliver aqueous outflow from the anterior chamber directly into Schlemm’s canal.

After phacoemulsification, the iStent infinite injector was guided under gonioscopic visualization to the nasal trabecular meshwork, where the first stent was implanted through the meshwork into Schlemm’s canal. In total, three stents were implanted internally over at least 4 clock hours of the anterior chamber angle, each approximately 2 clock hours from the previous. In some cases, after the second stent implantation, the surgeon exited the eye and repositioned in order to optimize the positioning and angle of approach. After implantation, the stent location and position were gonioscopically confirmed, then the surgeon removed viscoelastic and ensured corneal incision closure.

After surgery, patients used a topical non-steroidal anti-inflammatory drop and a topical antibiotic, with instructions to instill the medications over one to four weeks according to each surgeon’s standard postoperative administration and taper regimen.

### 2.3. Stastical Analysis

Statistical analysis employed a commercially available statistical software package (IBM SPSS Statistics for Windows, Version 20.0). Outcomes were given as descriptive statistics including the number of observations, mean, and standard deviation, unless otherwise indicated. Categorical measures were summarized with percentage and number of eyes. Postoperative IOP and medications at Month 12 versus baseline were compared using paired *t*-tests. Changes in proportional outcomes were analyzed using the McNemar test. Statistical significance was defined as a *p*-value of  <0.05.

## 3. Results

### 3.1. Study Poopulation

The preoperative demographic and ocular characteristics of this 233-eye cohort are provided in Table 1. The mean age was 73.3 years and most patients were female (59.7%). The majority had POAG (90.2%), most eyes were either mild (62.2%) or moderate (34.4%) in severity, and 17.7% of eyes had a history of prior SLT. Eyes had a preoperative baseline mean IOP of 16.9 ± 5.0 mmHg, were on 1.26 ± 0.94 mean medications and had a baseline visual field mean deviation of −3.7 ± 4.9 dB. Structurally, eyes had a mean retinal nerve fiber layer (RNFL) thickness of 78.6 ± 20.2 µm on OCT, and a corneal thickness of 541.0 ± 38.2 µm.

### 3.2. Efficacy

**Figure 1:**
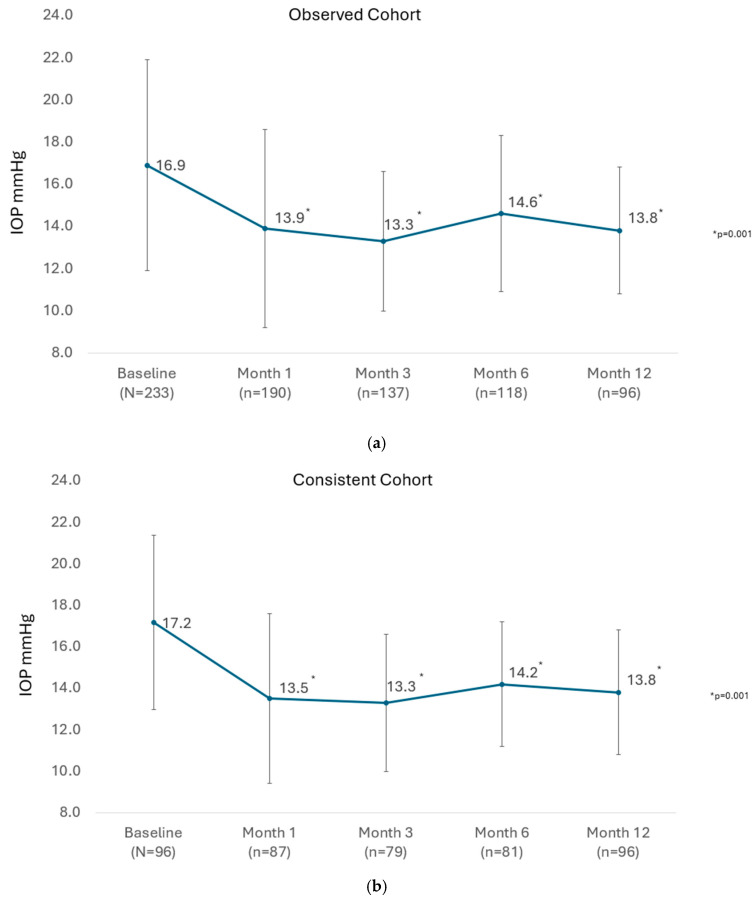
(**a**) Mean IOP over time in the observed cohort at each visit through 12 months. Foot-notes: IOP, intraocular pressure; *p*-value for paired *t*-test. (**b**) Mean IOP over time in the consistent cohort at each visit through 12 months. Footnotes: IOP, intraocular pressure; *p*-value for paired *t*-test.

The mean IOP of all eyes available at each visit until Month 12 in the observed cohort is presented in Figure 1a. There was a significant reduction in the mean IOP from 16.9 ± 5.0 mmHg preoperatively to between 13.3 ± 3.3 and 14.6 ± 3.7 mmHg at study visits from 1 month through 12 months (*p* = 0.001 at every post-operative visit to Month 12). In the consistent cohort of eyes with 12-month data (n = 96), the mean IOP was significantly reduced from 17.2 ± 4.2 mmHg preoperatively to 13.8 ± 3.0 mmHg at Month 12 (*p* = 0.001), as shown in Figure 1b.

**Figure 2:**
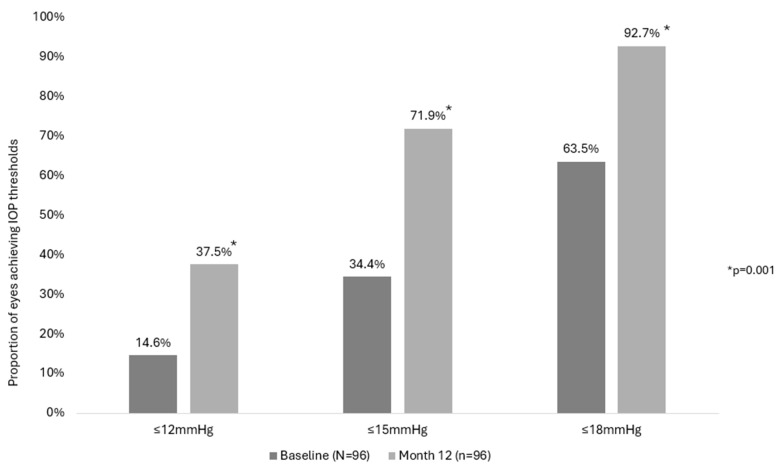
Proportion in consistent cohort achieving IOP thresholds (IOP ≤ 12 mmHg, ≤15 mmHg, ≤18 mmHg) at Baseline and 12 months. Footnotes: IOP, intraocular pressure; *p*-value for McNemar test.

In the observed cohort, the proportion of eyes achieving the prespecified target IOP cutoffs of ≤18 mmHg, ≤15 mmHg, and ≤12 mmHg increased from 66.4%, 40.9%, and 20.3% preoperatively to 92.7%, 71.9%, and 37.5%, respectively at Month 12, (all *p* = 0.001). Similarly, the proportion of eyes in the consistent cohort achieving the prespecified target IOP cutoffs of ≤18 mmHg, ≤15 mmHg, and ≤12 mmHg increased from 63.5%, 34.4%, and 14.6% preoperatively to 92.7%, 71.9%, and 37.5%, respectively, at Month 12, (all *p* = 0.001), as shown in Figure 2.

**Figure 3:**
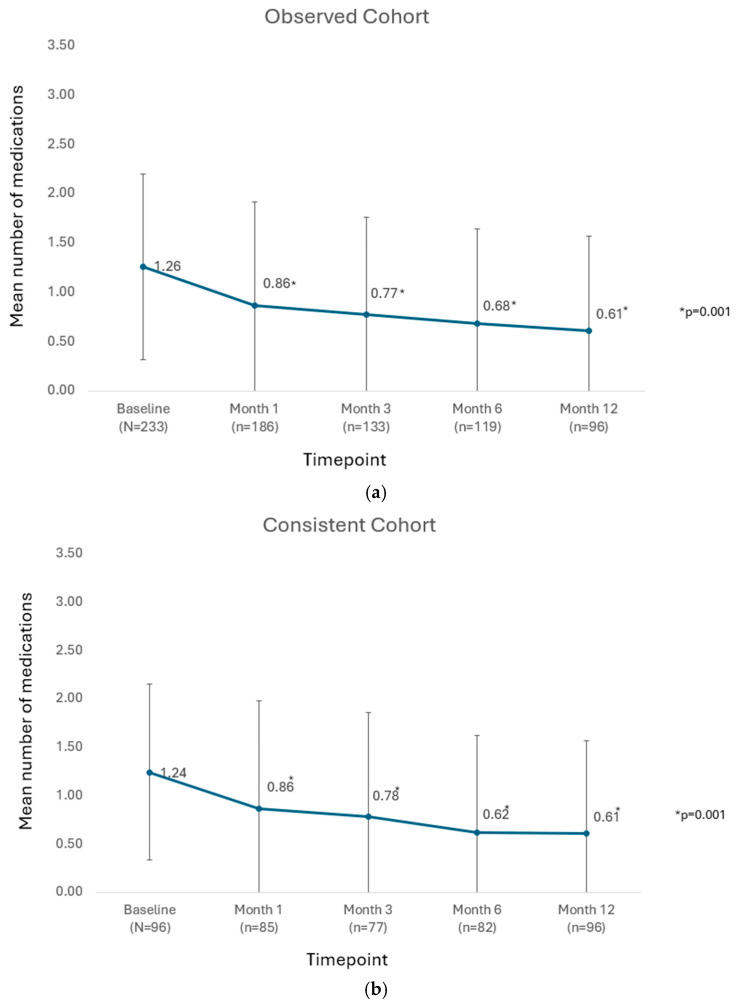
(**a**) Mean number of medications used by the observed cohort at each visit through 12 months. Footnotes: *p*-value for paired *t*-test. (**b**) Mean number of medications used by the consistent cohort at each visit through 12 months. Footnotes: *p*-value for paired *t*-test.

In the observed cohort of all available eyes at each postoperative timepoint, the mean number of medications was significantly reduced from 1.26 ± 0.94 preoperatively to between 0.61 ± 0.96 and 0.86 ± 1.05 mmHg at visits from Month 1 through Month 12 (*p* = 0.001 throughout), as shown in Figure 3a. Similarly in the consistent cohort, the mean number of medications was significantly reduced from 1.24 ± 0.91 medications preoperatively to 0.61 ± 0.96 medications at postoperative Month 12 (*p* = 0.001), as shown in Figure 3b.

**Figure 4:**
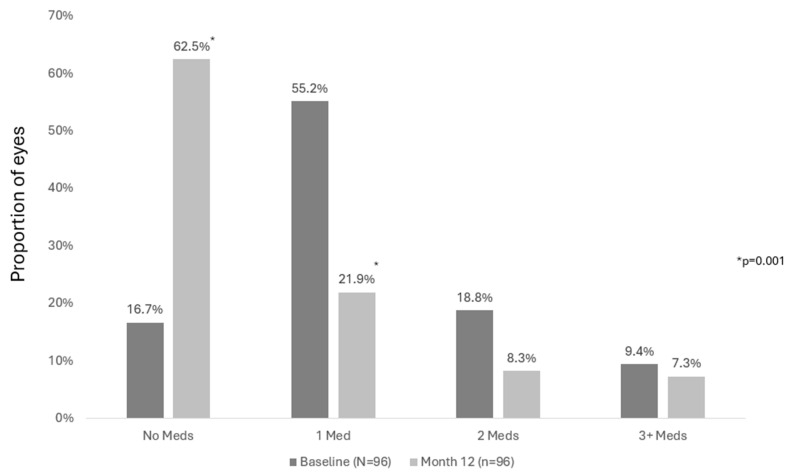
Proportion of eyes in consistent cohort that required 0, 1, 2 and 3 or more topical medications to manage IOP preoperatively and at postoperative Month 12. Footnotes: IOP, intraocular pressure; *p*-value for McNemar test.

For eyes in the observed cohort, the proportion of eyes that required no medications increased from 16.3% preoperatively to 62.5% (*p* = 0.001) postoperatively, and the proportion requiring one medication decreased from 55.8% to 21.9% (*p* = 0.001). The proportions of eyes on 2 or ≥3 medications reduced from 17.2% to 8.3% (*p* = 0.059) and 10.7% to 7.3% (*p* = 0.452), respectively. Results were similar in the consistent cohort at 12 months vs. preoperatively, as shown in Figure 4. In the consistent cohort, the proportion of eyes that required no medications increased from 16.7% preoperatively to 62.5% (*p* = 0.001) postoperatively, and the proportion requiring one medication decreased from 55.2% to 21.9% (*p* = 0.001). The proportions of eyes on 2 or ≥3 medications reduced from 18.8% to 8.3% (*p* = 0.058) and from 9.4% to 7.3% (*p* = 0.794), respectively. At Month 12 compared to preoperatively, 50.0% of eyes had fewer topical medications, 46.9% maintained their medications, and 3.1% increased their medications.

### 3.3. Safety

Safety measures consisted of complications, adverse events, and additional glaucoma surgeries. All eyes underwent successful phacoemulsification cataract surgery and the implantation of three iStent infinite stents. There were no reported intraoperative complications. Device-related postoperative adverse events were generally mild and transient; all events occurred within one week postoperative and resolved by one month postoperatively. Namely, seven eyes experienced IOP elevation at day 1 or week 1; six of these cases resolved with topical medications and one resolved with topical medications and anterior chamber tap. One eye had a clot in the nasal angle on day 1m which subsequently resolved without intervention or complications. Also on day 1, two eyes had hyphema and one eye had mild corneal edema; all three cases resolved by week 1.

Three eyes (<2% of cohort) underwent secondary glaucoma procedure(s) due to IOP and/or medications that were considered to be above target. One eye had Xen gel stent implantation at 6 months postoperatively due to a desire for further IOP and medication reduction (IOP/medications were 18 mmHg/3 medications postoperatively versus 20 mmHg/4 medications preoperatively). Two eyes had SLT: one at 6 months to decrease IOP (IOP/medications were 22 mmHg/3 medications postoperatively versus 18 mmHg/3 medications preoperatively), and the other eye at 12 months to decrease medication burden (IOP/medications were 11 mmHg/4 medications preoperatively versus 22 mmHg/2 medications preoperatively).

## 4. Discussion

This multicenter real-world study evaluated the iStent infinite trabecular micro-bypass combined with phacoemulsification. The study included consecutive patients of all glaucoma subtypes and severities from four surgical practices in the United States. By including a broad patient group and describing the outcomes associated with concomitant procedures performed in day-to-day practice, these results may be more aligned with real-word experience than those reported within the rigid structure and inclusion criteria of a clinical trial setting.

The observed mean reductions in IOP from baseline in both the observed cohort and consistent cohort were statistically significant at every post-operative visit through the 12-month follow-up period. The increase in the proportion of eyes achieving lower IOP thresholds at Month 12 versus preoperatively was also statistically significant. Overall, there were significant reductions in the postoperative mean and proportional medication reductions at each post-operative timepoint following iStent infinite implantation, with nearly all eyes (~97%) reducing or maintaining their preoperative medication and a fourfold increase in the proportion of eyes requiring no medications.

Within real-world practice, the limitations associated with topical therapy in glaucoma are well recognized. The challenges and complexity of daily topical treatment administration have been shown to negatively impact adherence to treatment, which increases the risk of glaucoma progression [18,19]. Studies have also demonstrated the burden of the associated costs of ongoing topical therapy, inadequate corneal penetration, the occurrence of ocular surface disease, IOP fluctuations, and a decreased quality of life [20]. Furthermore, due to certain comorbidities or contraindications due to interactions with systemic medications, there are some patients who are unable to use topical medications to manage IOP [21]. For these reasons, the reduction in topical medication use is significant, both for the clinical progress of the disease and for the patient.

Early procedural interventions provide the option to alleviate the burdens and challenges of topical therapy, and address the underlying physiological mechanisms early in the disease process, limiting progression and poor visual outcomes [22,23]. Also, the implementation of a procedural approach to glaucoma management delivers additional benefits, including more consistent IOP control, fewer side effects related to medication(s), lower overall long-term costs, a lower risk of disease progression, and freedom from reliance on patient adherence [20,24]. Significant improvements in quality of life with reduced topical medication use, reduced side effects, and a significant improvement in ocular surface health have also been well-documented [25,26].

As with any retrospective, real-world study, certain limitations should be acknowledged. All IOP measurements were taken as part of normal clinical care, and diurnal IOP measurements were not assessed. Instead of being determined by a controlled protocol, medication use was guided according to the surgeons’ usual clinical practice. Cases were completed in combination with cataract surgery, so it was not possible to delineate the potentially IOP-lowering effect of lens extraction from that of iStent infinite implantation. Due to the relatively recent availability of iStent infinite, current results to Month 12 include 96 of the 233 that received iStent infinite; however, this was remedied by presenting both the consistent and observed cohorts. These patients will continue to be followed and outcomes for the larger cohort over longer periods will be possible in the future.

## 5. Conclusions

In conclusion, this study provides clinically relevant, real-world results that demonstrate significant IOP and medication reductions following iStent infinite trabecular micro-bypass and phacoemulsification. By including data from four different surgical sites, and by not excluding any glaucoma subtypes or severities, we think this study cohort can be readily generalizable to patients in actual practice. To our knowledge, the study constitutes the first real-world multicenter publication of iStent infinite with cataract surgery in the literature to date.

## Figures and Tables

**Table 1:** Summary of subject baseline demographic and clinical characteristics (N = 233).

Parameter, Statistic	Total (N = 233)
**Age, n (years)**	
Mean (SD)	73.3 (7.4)
**Gender, n (%)** Male Female	94 (40.3%) 139 (59.7%)
**Glaucoma Subtype, n (%)** Primary open-angle Glaucoma Mixed Mechanism Glaucoma Normal/Low Tension Glaucoma Traumatic Glaucoma	216 (92.7%) 9 (3.9%) 7 (3.0%) 1 (0.4%)
**Glaucoma Severity ^1^, n (%)** Mild Moderate Severe Indeterminate	112 (62.2%) 62 (34.4%) 5 (2.8%) 1 (0.6%)
**Prior Surgery ^1^, n (%)** None Selective Laser Trabeculoplasty LASIK Laser Peripheral Iridotomy Peripheral Iridotomy, and Laser for Retinal Lesions Panretinal Photocoagulation	139 (77.2%) 32 (17.7%) 5 (2.8%) 2 (1.1%) 1 (0.6%) 1 (0.6%)
**Preoperative intraocular pressure (IOP), mmHg** Mean (SD)	16.9 (5.0)
**Number of Medications at Baseline** Mean (SD)	1.26 (0.94)
**Cup to Disc Ratio** Mean (SD)	0.6 (0.4)
**Visual Field MD, dB** Mean (SD)	−3.7 (4.9)
**Retinal Nerve Fiber Layer (RNFL) thickness, µm** Mean (SD)	78.6 (20.2)
**Corneal Thickness, µm** Mean (SD)	541.0 (38.2)

N: entire study cohort sample size; n: number of eyes with each parameter; dB: decibels; IOP: intraocular pressure; mmHg, millimeters of mercury; µm: microns; SD: Standard deviation. ^1^ recorded for 180.

## Data Availability

The datasets generated during and/or analyzed during the current study are available from the corresponding author on reasonable request.
